# Bamboo anthropomorphism and sustainable behavioral intention: evidence from bamboo as a substitute for plastic in China

**DOI:** 10.3389/fpsyg.2026.1948458

**Published:** 2026-09-29

**Authors:** Yaosheng Zhang, Yiping Luo

**Affiliations:** Art Design Department, Shangyu College, Shaoxing University, Shaoxing, China

**Keywords:** bamboo anthropomorphism, bamboo as a substitute for plastic, environmental guilt, place attachment, regional differences, social norms, sustainable behavioral intention

## Abstract

**Background:**

Bamboo, as a sustainable alternative to plastic, offers nature-based solutions to global plastic pollution and public health crises, advancing the United Nations Sustainable Development Goals (SDGs). However, the cognitive mechanisms of the public's sustainable behavioral intention toward bamboo as a substitute for plastic are still not well known.

**Methods:**

Drawing on the Hierarchy of Effects (HOE) model, this study proposes an integrative cognition–affect–intention framework in which bamboo anthropomorphism is associated with sustainable behavioral intention through place attachment and environmental guilt. Structural equation modeling (SEM) and multi-group analysis (MGA) were conducted using 926 valid responses collected from 31 provincial-level administrative regions in China. Regional differences and social norms are added as grouping variables to assess cross-group stability and heterogeneity of the structural relationships.

**Results:**

Bamboo anthropomorphism was not significantly directly associated with sustainable behavioral intention. Significant indirect associations were observed through place attachment, environmental guilt, and their sequential mediation, with the indirect effect through environmental guilt being the largest. Multi-group analyses indicated overall structural stability, with exploratory path-specific differences across regional and social-norm groups.

**Conclusion:**

This study refines the application of the HOE model by distinguishing place-based relational affect from self-conscious moral emotion within the cognition–affect–intention process. The findings suggest that bamboo anthropomorphism is associated with sustainable behavioral intention primarily through indirect affective and moral pathways. Exploratory group comparisons further suggest pathway-specific contextual heterogeneity, providing preliminary implications for differentiated environmental communication promoting bamboo-based plastic substitution and supporting the SDGs.

## 1 Introduction

Global environmental degradation and climate change have intensified the urgent need for systemic transitions toward sustainability while drawing increasing attention to how individuals perceive, evaluate, and respond to environmental challenges. Among these challenges, plastic pollution has emerged as a critical global issue, directly implicating SDG 12 (Responsible Consumption and Production), SDG 13 (Climate Action), and SDG 15 (Life on Land). Without intervention, mismanaged plastic waste is projected to reach 121 million metric tons (Mt) annually by 2050 (Pottinger et al., 2024). Critically, over 90% of global plastic raw materials derive from fossil fuels, and plastics account for 4.5% of global carbon emissions (Cabernard et al., 2021; Stegmann et al., 2022). These escalating environmental pressures highlight not only the urgent need to develop sustainable alternatives to plastic but also the importance of understanding the psychological and cognitive processes that shape public engagement with sustainable material substitution.

China is a major bamboo producer and possesses unique advantages in advancing bamboo-based alternatives to plastic. First, China has one of the world's richest bamboo resources, comprising 861 species and infraspecific taxa across 43 genera, with a total bamboo forest area of 6.41 million hectares (China Bamboo Industry Association, 2022; Liu et al., 2018). This abundant resource base provides a stable supply of renewable raw materials for large-scale bamboo substitution. Second, bamboo grows rapidly and is natural, biodegradable, and environmentally friendly, making it well suited to replacing conventional plastics (Li et al., 2025). Bamboo products may also generate substantially lower, or even negative, lifecycle carbon emissions compared with plastic products (Zhang et al., 2025). For example, previous research reported a carbon footprint of −14.89 kg CO_2_-eq/m^3^ for bamboo scrimber flooring (Gu et al., 2019). Using the experimentally measured air-dried density of 1,204 kg/m^3^ (Liu et al., 2021), this converts to approximately −0.0124 kg CO_2_-eq per kg of flooring. On the same mass basis, PVC production generates approximately 7.83 kg CO_2_-eq per kg (Liang and Yu, 2023). In addition, China has developed a broad technological and industrial foundation for applying bamboo in packaging, furniture, engineering materials, sports equipment, automotive interiors, decoration, and protective equipment (Ye et al., 2024; Zhang H. et al., 2024; Zhang M. et al., 2024). Taken together, China's abundant bamboo resources, favorable environmental performance, and expanding industrial applications give the country a strong comparative advantage in promoting bamboo as a strategic substitute for plastic and supporting the transition toward more sustainable material systems.

As a global initiative to combat plastic pollution and advance public health, the concept of “bamboo as a substitute for plastic” has evolved from a theoretical proposal into concrete action. For instance, in the Philippines, bamboo frame buildings provide an economical and low-carbon strategy for social housing projects (Bundi et al., 2024). In Ethiopia, bamboo generates income for local communities and is widely applied across construction, furniture manufacturing, food processing, medicinal preparation, and musical instrument production (Abebe et al., 2022). In China, the bamboo industry boasts an annual output value exceeding 520 billion Chinese Yuan (CNY), with over 15,000 types of bamboo products. Currently, there are more than 10,000 bamboo processing enterprises, and the bamboo industry chain employs over 29 million people (English.gov.cn, 2026). However, the success of material-substitution initiatives ultimately depends partly on the public's willingness to adopt bamboo-based products and practices. Although “bamboo as a substitute for plastic” has made notable advances in the upstream supply chain, the psychological mechanisms driving the public's sustainable behavioral intention toward bamboo as a substitute for plastic remain underexplored (Ji et al., 2025), limiting its broad adoption.

This issue reflects a broader gap in research on the cognitive foundations of sustainable decision-making. Existing research on “bamboo as a substitute for plastic” has primarily focused on industrial economic analysis, technical performance evaluation, and consumer perceptions of bamboo products—largely confined to price, quality, and design attributes (Ji et al., 2025; Liu et al., 2025; Wang et al., 2025; Wei et al., 2023, 2025; Xue et al., 2024; Zhao et al., 2024), while paying limited attention to the cognitive processes through which individuals interpret and evaluate bamboo as a sustainable substitute for plastic. Importantly, cognitive appraisals do not operate in isolation but can activate affective and moral responses and are further shaped by contextual influences, jointly guiding sustainable behavioral intention (Liu et al., 2020; Yin et al., 2025). Yet, how these interconnected processes shape sustainable behavioral intention toward bamboo as a substitute for plastic remains insufficiently understood.

Anthropomorphism is the cognitive appraisal process of attributing human characteristics, emotions, or intentions to non-human entities (Alabed et al., 2022; Ding et al., 2022; Yang et al., 2020). It offers a novel theoretical lens to address the research gap. In marketing and environmental communication, existing studies show that anthropomorphism significantly enhances public emotional resonance and fosters sustainable behavioral intention (He and Shan, 2022; Jain et al., 2022; Lin et al., 2026; Liu et al., 2024; Qin et al., 2025). In China, bamboo is deeply tied to cultural identity. Its 7,000-year history of craftsmanship has fostered a rich tradition of bamboo anthropomorphism in art, literature, and daily life (Zhang and Zhang, 2019). Building on this cultural and theoretical foundation, the present study adopts bamboo anthropomorphism as the independent variable.

To clarify how bamboo anthropomorphism shapes the public's sustainable intention toward bamboo as a substitute for plastic, this study constructs a theoretical framework based on the Hierarchy-of-Effects (HOE) model. The model describes a sequential process from cognition through affect to sustainable behavioral intention (Lavidge and Steiner, 1961). Accordingly, in the present study, bamboo anthropomorphism is treated as a cognitive appraisal. Place attachment is conceptualized as a relational and place-based affective response, whereas environmental guilt is conceptualized as a self-conscious moral emotion. These mechanisms may independently and sequentially translate anthropomorphic cognition into sustainable behavioral intention toward bamboo as a substitute for plastic.

Moreover, the relationships within the proposed cognition–affect–intention framework may vary across contextual settings. In China, substantial north–south differences in bamboo resource distribution and the development of the bamboo-processing industry (China Bamboo Industry Association, 2022) provide a contextual basis for examining whether the structural relationships differ across regional groups. Social norms, as an important external influence on individual behavior (Cialdini et al., 1990), may likewise be associated with variation in these relationships across normative contexts. Accordingly, regional differences and social norms are incorporated as contextual grouping variables in the multi-group analysis to assess the cross-group stability of the proposed model and to explore possible structural heterogeneity. This approach provides a basis for evaluating the generalizability of the proposed relationships across different regional and social-norm contexts.

In summary, this study aims to answer two questions using structural equation modeling (SEM) and multi-group analysis (MGA). First, how is bamboo anthropomorphism related to the public's sustainable behavioral intention toward bamboo as a substitute for plastic in China? Second, does the overall pattern of structural relationships within the proposed cognition–affect–intention model differ across regional and social-norm groups?

The main contributions and novelties of the present study are as follows:

(1) Proposing an integrative cognition–affect–intention mechanism. This study applies and refines the HOE framework in the context of bamboo-based plastic substitution by positioning bamboo anthropomorphism as the cognitive starting point and distinguishing place attachment from environmental guilt as two theoretically distinct mechanisms within the affective stage. Regional differences and social norms are further incorporated as contextual grouping variables to assess the cross-group stability and potential heterogeneity of the proposed structural relationships. This framework provides a systematic explanation of the public's sustainable behavioral intention toward bamboo as a substitute for plastic beyond conventional product-attribute evaluations.
(2) Demonstrating the relationship between bamboo anthropomorphism and sustainable behavioral intention toward bamboo as a substitute for plastic. The findings reveal significant indirect associations between bamboo anthropomorphism and sustainable behavioral intention through place attachment, environmental guilt, and their serial mediating effects, thereby elucidating the mediating roles of affective and moral factors within the integrative cognitive mechanism.
(3) Providing new insights into contextual heterogeneity. This study examines the cross-group stability of the integrative cognition–affect–intention framework across regional and social-norm contexts. The omnibus multi-group analyses indicated overall structural stability across both grouping variables. Exploratory follow-up comparisons nevertheless suggested localized differences in the EG → SBI relationship across regional groups and in the BA → PA relationship across social-norm groups. These findings indicate that any contextual heterogeneity may be pathway-specific rather than characteristic of the structural model as a whole, although the localized patterns should be regarded as exploratory rather than confirmatory.

## 2 Literature review

### 2.1 Hierarchy of effects model

A body of research has been conducted to study the formation process of an individual's sustainable behavioral intention according to the HOE model, which suggests that external stimuli induce three successive psychological stages: cognition, affect, and intention (Lavidge and Steiner, 1961). Studies applying this framework have examined independent variables such as the perceived impacts of rural tourism (Li et al., 2020), the need for cognition (Paredes et al., 2023), social media parameters (Sharma et al., 2022), and the perceived CSR information substantiality (Hazel and Kang, 2018). Methodologically, researchers have employed SEM (Li et al., 2020), PLS-SEM (Paredes et al., 2023), Neural Network (NN) analysis (Sharma et al., 2022), and causal quantitative research (Pérez and De Los, 2023), with samples covering the new generation of farmers (Li et al., 2020), millennials (Paredes et al., 2023), and young consumers (Sharma et al., 2022). The model therefore provides a useful foundation for examining how a sustainability-related stimulus is psychologically processed before it affects behavioral intention.

Notably, existing research on bamboo-based product adoption behavioral intention primarily focuses on the linear influence of cognitive factors, such as product quality, product creativity, and eco-friendly attributes (Shi et al., 2025; Xue et al., 2024). Consequently, relatively less attention has been paid to the mechanisms driven by emotional connections and symbolic meanings associated with the products. Drawing on prior HOE research (Lavidge and Steiner, 1961; Wan and Chen, 2021; Williams et al., 2021), during the “cognition” stage, bamboo anthropomorphism may be positively associated with individuals' environmental awareness through perceived human-like traits and may thereby positively influence their sustainable behavioral intention toward bamboo as a substitute for plastic. At the “affect” stage, place attachment and environmental guilt may serve as key mediating variables linking bamboo anthropomorphism to the public's sustainable behavioral intention (Daryanto and Song, 2021; Yang et al., 2023). It is still unknown whether the above factors are direct mediators or whether they operate in a sequence to connect bamboo anthropomorphism and sustainable behavioral intention. Future research should investigate the specific pathways and effect sizes of the aforementioned mediation effects.

The application of the HOE model to bamboo-based substitution nevertheless requires refinement. First, the model has rarely been examined in a culturally embedded sustainable material-substitution context in China. Second, the affective stage is often treated as a single broad category, although relational attachment and self-conscious moral emotion may perform different functions. Third, contextual conditions may be associated with variation across different paths of the cognition-affect-intention process. These limitations motivate an expanded, context-sensitive HOE framework.

Accordingly, in the present study, the HOE model is used as an organizing framework for distinguishing the broad stages through which bamboo-related cognition may be translated into sustainable behavioral intention, rather than as a specification of a single mandatory causal chain. Bamboo anthropomorphism is positioned at the cognitive stage, while place attachment and environmental guilt represent two theoretically distinguishable responses within the affective stage, and sustainable behavioral intention represents the intention stage. Building on this structure, the empirical model examines both parallel and sequential mediating pathways to capture the possibility that affective responses may operate independently or in sequence within the broader cognition–affect–intention process. The direct path from bamboo anthropomorphism to sustainable behavioral intention is also retained to examine whether anthropomorphic cognition is associated with intention independently of the proposed affective mechanisms. In addition, regional differences and social norms are incorporated as contextual grouping variables to assess whether the structural relationships within the cognition–affect–intention model vary across different regional and normative contexts.

### 2.2 Anthropomorphism and sustainable behavioral intention

Another strand of literature explores the relationship between anthropomorphism and sustainable behavioral intention, with prior studies generally reporting a positive association. For example, research has shown that anthropomorphic strategies have improved consumers' recycling attitudes and behavioral intentions (Jain et al., 2022). Sestino and D'Angelo (2023) pointed out that perceived anthropomorphism was positively associated with individuals' intention to use digital-based healthcare services. Similarly, Wang and Park concluded that anthropomorphism was positively related to willingness to purchase insect foods via the mediating effect of psychological closeness (Wang and Park, 2023). A study revealed that green brand anthropomorphism was positively associated with consumers' repurchase intention (Sehgal et al., 2023). These cross-field studies validate the effectiveness of anthropomorphism in explaining and guiding individuals' sustainable behavioral intention.

Although anthropomorphism can enhance public engagement with environmental issues by endowing elements with human-like traits (He and Shan, 2022; Lu et al., 2021; Qin et al., 2025), its potential role in promoting “bamboo as a substitute for plastic” is understudied. Research in this area focuses on industrial and technical aspects (Lin et al., 2022; Liu et al., 2025; Zhao et al., 2024), with little attention to whether and how bamboo anthropomorphism is associated with the public's sustainable behavioral intention toward bamboo as a substitute for plastic.

Despite the growing evidence supporting the effectiveness of anthropomorphism, several research gaps remain. First, although anthropomorphism has been examined across diverse contexts such as brands, products, digital services, and environmental communication, little attention has been paid to culturally symbolic natural resources. Consequently, whether bamboo anthropomorphism—a culturally significant symbol in China—can effectively promote sustainable behavioral intention toward bamboo as a substitute for plastic remains unclear. Second, existing studies predominantly focus on average treatment effects, providing limited evidence on potential heterogeneity across different populations. In the Chinese context, regional cultural differences between northern and southern residents may influence how anthropomorphic bamboo is perceived and whether such appeals translate into stronger sustainable behavioral intention. Addressing these gaps would not only extend anthropomorphism theory to a culturally grounded context but also provide practical guidance for designing more targeted communication strategies for promoting bamboo as a substitute for plastic.

## 3 Research hypotheses

### 3.1 Bamboo Anthropomorphism (BA) and Sustainable Behavioral Intention (SBI) toward bamboo as a substitute for plastic

Anthropomorphism involves attributing human characteristics to non-human entities, thereby endowing them with human-like attributes (Alabed et al., 2022; Ding et al., 2022; Yang et al., 2020). In the present study, bamboo anthropomorphism refers specifically to the cognitive attribution of human-like mental capacities to bamboo, including consciousness, awareness, emotional experience, and free will. As a cognitive appraisal, bamboo anthropomorphism may foster the public's sustainable behavioral intention toward bamboo as a substitute for plastic through the two linked channels described below. First, anthropomorphic cues may reduce psychological distance and increase perceived connectedness with bamboo. Anthropomorphism of nature can increase a person's sense of connection with nature and thus encourage pro-environmental behavior (Yang et al., 2023). Bamboo anthropomorphism may strengthen the emotional connection between people and bamboo, making the role of bamboo as a plastic substitute more familiar and acceptable. Second, anthropomorphism may be positively associated with the perceived warmth, familiarity, and symbolic value of bamboo-based alternatives, thereby strengthening adoption intentions. Brand anthropomorphism has been shown to increase the intention to engage in green consumption in the consumption context by raising both perceived green quality and perceived green value (Nie et al., 2024). By analogy, anthropomorphism for bamboo products may make them more relatable to people, and thus improve their image as environmentally friendly and high-quality; consequently, it can promote the public's sustainable behavioral intention. Thus, the following hypothesis is proposed.

*H1:* BA is positively associated with the public's SBI.

### 3.2 The mediating role of Place Attachment (PA)

Place attachment refers to the cognitive and emotional bond between individuals and a specific place (Hidalgo and Hernández, 2001; Jia et al., 2025), and generally consists of two aspects: place identification and place dependence (Chang et al., 2023; Nursyamsiah and Setiawan, 2023). Bamboo anthropomorphism is theoretically expected to be positively associated with place attachment in three ways. First, anthropomorphism is an effective way to reduce psychological distance. It increases emotional resonance and identification among humans, non-human entities, and their surroundings (Berenguer, 2010; Jayakody et al., 2024). Thus, bamboo anthropomorphism can strengthen place attachment by deepening people's emotional identification with bamboo, which extends to its environment. Second, bamboo anthropomorphism enhances cultural identification with the place. Place attachment reflects an individual's sense of belonging and judgment of a place (Moore and Graefe, 1994). Bamboo holds deep historical and symbolic meaning in Chinese culture (Dlamini et al., 2022). Consequently, anthropomorphism transforms bamboo from a natural element into a tangible carrier of cultural symbols, promoting public identification with regional culture and strengthening place attachment. Third, anthropomorphism increases human-place similarity (Tam et al., 2013), enabling individuals to more easily establish connections among themselves, bamboo, and their environment, thereby supporting the development of place attachment. Therefore, the following hypothesis is proposed:

*H2:* BA is positively associated with PA.

Place attachment is a key predictor of sustainable behavior. For example, Daryanto and Song (2021) found that place attachment positively affects pro-environmental behavior. Another study found that place attachment boosts an individual's perceived environmental responsibility (Soopramanien et al., 2023). When bamboo anthropomorphism fosters strong place attachment, the public's attention to the local environment will increase, and they will be more inclined to choose bamboo-based alternatives to reduce plastic pollution. Therefore, we propose the following hypotheses:

*H3:* PA is positively associated with the public's SBI.
*H4:* PA mediates the relationship between the public's BA and SBI.

### 3.3 The mediating role of Environmental Guilt (EG)

Environmental guilt refers to the sense of remorse individuals feel for harming nature (Ferguson and Branscombe, 2010). This guilt motivates sustainable behaviors by making individuals aware of their group's responsibility for environmental harm and prompting them to take restorative action (Harth et al., 2013). For example, perceiving that group members have harmed the environment triggers feelings of environmental guilt. This guilt, in turn, motivates efforts to compensate (Moore and Yang, 2020; Rees et al., 2015). Thus, when the public experiences general environmental guilt in the bamboo-for-plastic context, they may develop a stronger intention to choose bamboo as a substitute for plastic to alleviate these negative emotions and fulfill their environmental responsibilities. Therefore, the following hypothesis is proposed:

*H5:* EG is positively associated with the public's SBI.

According to Yang et al., environmental guilt serves as a mediator between anthropomorphism of nature and pro-environmental behavior (Yang et al., 2023). By analogy, bamboo anthropomorphism fosters greater concern for bamboo and its natural environment (Tam, 2019), making people more prone to environmental guilt when plastic pollutes the environment. To alleviate this guilt, individuals are motivated to undertake restorative actions (Harth et al., 2013), for instance, forming a behavioral intention to adopt bamboo as a substitute for plastic. Therefore, we propose the following hypotheses:

*H6:* BA is positively associated with EG.
*H7:* EG mediates the relationship between BA and the public's SBI.

Place attachment enhances attention to environmental conditions (Kyle et al., 2005), and predicts the discrete emotion of guilt (Shipley et al., 2023). Therefore, when the environment is harmed, this attachment may transform into environmental guilt. Accordingly, the following hypothesis is proposed:

*H8:* PA is positively associated with EG.

Overall, bamboo anthropomorphism evokes environmental guilt, both directly and through place attachment, thereby increasing sustainable behavioral intention toward bamboo as a substitute for plastic. Therefore, we propose the following hypotheses:

*H9:* PA and EG sequentially mediate the relationship between the public's BA and SBI.

### 3.4 The contextual role of Regional Differences (RD) and Social Norms (SN)

There are obvious north-south differences in China's bamboo resources and processing industry (China Bamboo Industry Association, 2022). The eight provinces with the largest bamboo forest areas are Fujian, Jiangxi, Zhejiang, Hunan, Sichuan, Guangdong, Anhui, and Guangxi. Together, these provinces have 5.707 million hectares of bamboo, which is 89.02% of the nation's total. Their output in bamboo-based panels, daily necessities, flooring, and furniture accounts for 97.21, 99.43, 99.24, and 99.57% of the national total, respectively. All these provinces are in southern China. These patterns indicate a substantial geographic concentration of bamboo resources and bamboo-related industries in southern China.

Regional differences are therefore considered in this study as a broad contextual grouping variable rather than as a direct measure of bamboo-resource abundance. The geographic concentration of bamboo resources and bamboo-related industries provides the rationale for comparing respondents residing in southern and northern China using the Qinling–Huai River boundary. However, the binary North–South classification captures multiple dimensions of regional context and should not be interpreted as an equivalent measure of provincial bamboo forest area, bamboo-industry development, or individual exposure to bamboo resources. Accordingly, the regional analysis examines whether the proposed psychological relationships vary across broad geographic contexts rather than testing the causal effect of bamboo-resource endowment itself. Therefore, the following hypothesis is proposed:

*H10:* The overall pattern of structural relationships within the integrative cognition–affect–intention model differs between the northern and southern regional groups.

Social norms refer to behavioral expectations and guidelines that are collectively shared and followed by members of a community (Cialdini et al., 1990). China shows certain regional differences in the social norms that promote the concept of “bamboo as a substitute for plastic” (Ji et al., 2025). In fact, such norms are often the result of social pressure from government procurement policies, industry standards, community waste initiatives, and media promotion. Chinese society is relationship-oriented, and people are very concerned about what others think of them (Fei et al., 1992). With increased perceived social pressure, more people are conforming to the expectations of these reference groups (Hwang, 1987).

Under such conditions, the extent to which individual cognitive evaluations and affective responses translate into behavioral intentions may differ from that observed under weak normative pressure. Conversely, when perceived social norms are weak, individual judgments and emotional responses may play a relatively greater role in shaping intention.

Thus, social norms are conceptualized here not merely as an additional predictor of intention, but as a contextual condition under which the structural relationships within the cognition–affect–intention process may vary. Comparing respondents with relatively high vs. low perceived social norms therefore allows us to assess whether the proposed structural model operates similarly across different normative contexts. Thus, the following hypothesis is put forward.

*H11:* The overall pattern of structural relationships within the integrative cognition–affect–intention model differs between the low- and high-social-norm groups.

Based on these hypotheses, a conceptual model (Figure 1) was developed to examine the direct and indirect associations between bamboo anthropomorphism and sustainable behavioral intention toward bamboo as a substitute for plastic, together with the cross-group stability of the proposed structural relationships across regional and social-norm contexts.

**Figure 1:**
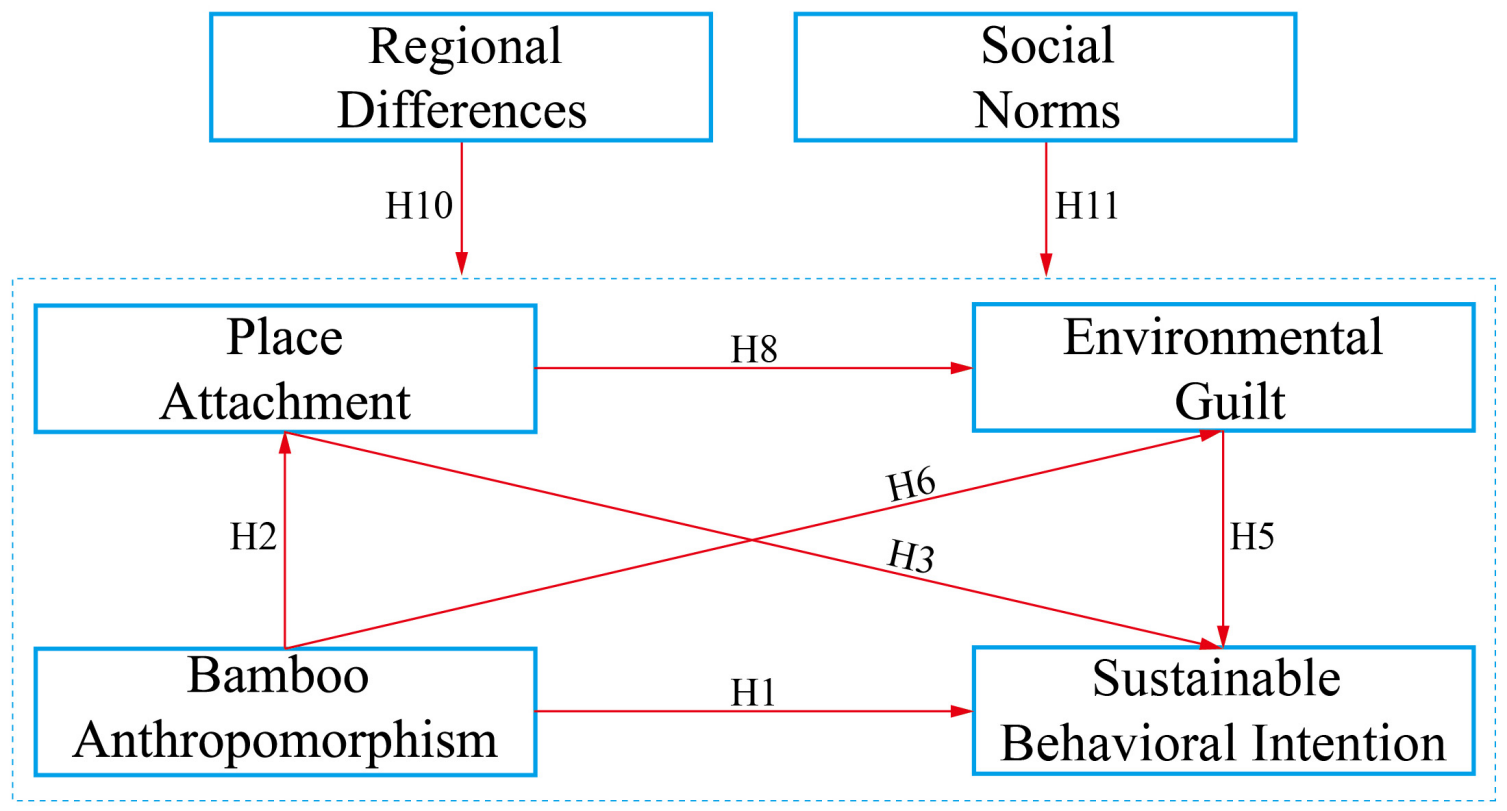
Conceptual model.

## 4 Methodology

### 4.1 Instruments and measurements

As shown in Table 1, all measurement items in this study were adapted from existing research scales. For bamboo anthropomorphism, four items measured the public's perception of bamboo anthropomorphism (e.g., “I believe bamboo has consciousness similar to that of humans”; “I believe bamboo is aware of its surroundings”), according to anthropomorphism of nature scales (Tam, 2014; Yang et al., 2023). For place attachment, six items measured place identification and place dependence (e.g., “The place I live means a lot to me”; “No other place can compare to where I live”), adapted from place attachment scales (Cheung and Hui, 2018; Raymond et al., 2010). For environmental guilt, five items assessed an individual's sense of guilt arising from human-induced environmental damage (e.g., “I feel bad when I think about the damage that pollution has caused to nature”), according to guilt scales (Eom et al., 2021; Moore and Yang, 2020). For social norms, three items (e.g., “I've noticed that people around me have already started using bamboo as a substitute for plastic.”) were adapted from social norms scales (Ru et al., 2018; Zhang and Wan, 2021). Finally, for sustainable behavioral intention, four items (e.g., “I plan to implement the practice of bamboo as a substitute for plastic in the future.”) were taken from behavioral intention scales (Rausch and Kopplin, 2021; Zhang et al., 2020).

**Table 1:** Measurement model results.

Construct	Items	Loading	Cronbach's α	CR	AVE
Bamboo Anthropomorphism (BA)	I believe bamboo has consciousness similar to that of humans.	0.805^***^	0.868	0.872	0.630
	I believe bamboo is aware of its surroundings.	0.808^***^			
	I believe bamboo has emotional experience.	0.830^***^			
	I think bamboo has its own free will.	0.727^***^			
Place Attachment (PA)	The place I live is very special to me.	0.858^***^	0.925	0.926	0.676
	I identify strongly with the place I live.	0.872^***^			
	The place I live means a lot to me.	0.794^***^			
	No other place can compare to where I live.	0.863^***^			
	I believe where I live suits my lifestyle better than anywhere else.	0.810^***^			
	I feel that the time I've spent where I live is irreplaceable.	0.728^***^			
Environmental Guilt (EG)	I feel bad when I think about the damage that pollution has caused to nature.	0.786^***^	0.863	0.866	0.564
	I easily feel guilty about the environmental damage caused by human activities.	0.779^***^			
	I feel regret for human pollution damage toward nature in the past.	0.744^***^			
	I believe we have a responsibility to help restore the damage that has been done to nature.	0.668^***^			
	When I think about the pollution damage humans have caused to nature, I feel sorry.	0.773^***^			
Social Norms (SN)	I've noticed that people around me have already started using bamboo as a substitute for plastic.	0.741^***^	0.815	0.818	0.601
	Those close to me suggest adopting bamboo as a substitute for plastic to reduce plastic usage.	0.815^***^			
	Many believe we should implement the practice of bamboo as a substitute for plastic.	0.767^***^			
Sustainable Behavioral Intention (SBI)	I am willing to adopt the sustainable practice of bamboo as a substitute for plastic.	0.845^***^	0.931	0.930	0.771
	I intend to start using bamboo products instead of plastic ones.	0.908^***^			
	I plan to implement the practice of bamboo as a substitute for plastic in the future.	0.906^***^			
	I am willing to promote the concept of bamboo as a substitute for plastic to others.	0.850^***^			

^***^Indicates *p* < 0.001.

A 7-point Likert scale was used to ask respondents how much they agreed with the following options, selecting a number from 1 (strongly disagree) to 7 (strongly agree). A small group of experts checked for errors in the questionnaire. This group consisted of 8 university faculty members and students. A small-scale trial with 50 participants was conducted to assess item clarity, and minor linguistic revisions were made accordingly. The questionnaire administered in the main survey initially contained 23 items. During subsequent scale refinement, one item was removed because its wording was not sufficiently aligned with the conceptual definition of the corresponding construct. The final measurement model therefore comprised 22 items.

Three additional design features should be noted. First, the opening section introduced the purpose of this survey to the participants. It explicitly defined “bamboo as a substitute for plastic” and “sustainable behavioral intention toward bamboo as a substitute for plastic”. Second, given China's geography, the 800-millimeter annual isohyets are divided into the North and the South. The Qinling Mountains, the Huai River, and the southeast coast of the Qinghai-Tibet Plateau are all on this line. The two areas are also listed in the questionnaire. Third, social norms were measured as a continuous construct. For the purpose of multi-group SEM, respondents were divided into low- and high-social-norm groups according to the sample mean of the social-norm score. This grouping was used to assess the cross-group stability of the overall structural model and should not be interpreted as implying that social norms are inherently categorical. Because dichotomization may result in information loss and range-restriction problems (MacCallum et al., 2002), the social-norm-based MGA results were interpreted cautiously.

### 4.2 Sampling and data collection

#### 4.2.1 Sample size consideration

For initial survey planning, the conventional Cochran formula for estimating a population proportion in a large population was used as a general recruitment benchmark (Cochran, 1977):


$$\begin{array}{l} n=\frac{Z^{2}p\left(1-p\right)}{e^{2}} \end{array}$$

A 95% confidence level (*Z* = 1.96), a conservative population proportion of *p* = 0.5, and a 5% margin of error (*e* = 0.05) were adopted, yielding a minimum required sample size of approximately 385 respondents. Because the present study employed non-probability online sampling and its primary analyses involved structural equation modeling and multi-group comparisons, this value was not treated as a formal SEM-based minimum sample-size requirement. Rather, it served only as a general benchmark for survey recruitment.

Given that the planned analyses included structural equation modeling and multi-group comparisons, the recruitment target was set substantially above this general benchmark to ensure adequate sample sizes for model estimation and subgroup comparisons. Additional recruitment also allowed for the exclusion of incomplete or low-quality responses during data screening. Ultimately, 1,100 respondents were recruited, of whom 926 valid responses were retained for the final analysis.

#### 4.2.2 Data collection

This study was conducted in accordance with the Declaration of Helsinki and applicable national legislation. Participation in the survey was voluntary. Before beginning the questionnaire, respondents were provided with information concerning the purpose of the study, the voluntary nature of participation, and the collection and use of their questionnaire responses. Adult participants provided electronic written informed consent through the online questionnaire platform before participation. For participants under 18 years of age, electronic written informed consent was obtained from a parent or legal guardian, and assent was obtained from the minor participant before participation. Only respondents who completed the applicable consent or assent procedure were permitted to proceed to the questionnaire.

Data were collected between November 2025 and January 2026. A total of 1,100 respondents were recruited through the Tencent Questionnaire platform. Respondents who completed the questionnaire and satisfied the response-quality criteria received 3 CNY as a small token of appreciation for their participation. The incentive was not used as an indicator of respondent engagement; instead, response quality was assessed through explicit screening procedures.

Specifically, the data were screened in three steps. First, two instructed-response attention-check items were embedded in the questionnaire. These items explicitly instructed respondents to select a specified response option (e.g., “Agree” or “Disagree”), allowing us to identify respondents who did not carefully read or follow the questionnaire instructions. A total of 94 responses were excluded for failing the two attention checks. Second, 58 responses were excluded because their completion times were below the predefined minimum threshold of 60 seconds. Third, 22 responses were excluded because they exhibited invariant or otherwise predefined invalid response patterns. After application of these quality-control criteria, 926 of the 1,100 collected responses were retained for analysis, corresponding to an analytic retention rate of 84.2% and an overall exclusion rate of 15.8%.

The final 926 valid samples were collected from 31 provincial-level administrative regions in China. There were 562 respondents from southern China and 364 from northern China. The provinces contributing the largest proportions of respondents were Guangdong (15.7%), Shandong (7.5%), and Zhejiang (6.8%). The geographic distribution is based on respondents' self-reported province. The detailed sample profile is presented in Table 2.

**Table 2:** Sample profile.

Item	Categories	Frequency	Percentage
Gender	Male	345	37.26%
	Female	581	62.74%
Age	0–17	6	0.65%
	18–24	345	37.26%
	25–45	517	55.83%
	46–60	54	5.83%
	60+	4	0.43%
Income (CNY)	0–2,000	144	15.55%
	2,001–3,000	120	12.96%
	3,001–5,000	245	26.46%
	5,001–8,000	245	26.46%
	8,001–10,000	104	11.23%
	10,000+	68	7.34%
Residence	The south	562	60.69%
	The north	364	39.31%

The survey was administered through the Tencent Questionnaire platform and was not designed as a probability sample calibrated to reproduce the national demographic distribution of China. No post-stratification or population weighting was applied. Compared with national population benchmarks from the Seventh National Population Census (National Bureau of Statistics of China, 2021), the present sample contained a higher proportion of females (62.74% vs. 48.76% nationally) and was concentrated among younger and middle-aged adults. Specifically, 93.09% of respondents were aged 18–45 years, whereas the national census reported that 63.35% of the population was aged 15–59 years. Because the age categories are not directly equivalent, this comparison should be interpreted descriptively rather than as an exact age-matched comparison. These demographic differences should be considered when interpreting the generalizability of the findings.

#### 4.2.3 Data analysis

The data were analyzed using SPSS 27.0 and AMOS 26.0. Prior to the main analyses, descriptive statistics, including means, standard deviations, skewness, and kurtosis, were calculated for all constructs. The final analytic dataset was examined for missing values, and no missing values were identified among the 926 valid responses; therefore, no missing-data imputation was required. Multivariate normality was assessed using the multivariate kurtosis statistic and its critical ratio (C.R.) provided by AMOS 26.0. The structural equation model was estimated using maximum likelihood (ML) estimation.

Model fit was evaluated using multiple complementary fit indices, including CMIN/DF, RMSEA, SRMR, GFI, AGFI, NFI, IFI, CFI, and TLI, where applicable. Following established recommendations, particular attention was given to RMSEA, SRMR, CFI, and TLI. Values close to or below 0.06 for RMSEA and 0.08 for SRMR, and values close to or above 0.95 for CFI and TLI, were considered indicative of relatively good fit (Hu and Bentler, 1999). These cutoff values were treated as guidelines rather than rigid decision rules, and model fit was evaluated based on the overall pattern of multiple indices rather than any single statistic (Hu and Bentler, 1999; Kline, 2023).

First, potential common method bias (CMB) was assessed using an unmeasured latent method construct (ULMC) approach. Second, the evaluation of reliability and construct validity was conducted through Cronbach's α, exploratory factor analysis (EFA), confirmatory factor analysis (CFA), composite reliability (CR), and average variance extracted (AVE). The evaluation criteria aligned with established recommendations by Kline (2023), Hair et al. (2019), and (Hu and Bentler 1999). Next, SEM was employed to estimate the hypothesized relationships among bamboo anthropomorphism, place attachment, environmental guilt and sustainable behavioral intention (Hair et al., 2019; Kline, 2023). Then, bias-corrected bootstrapping with 5,000 resamples and 95% confidence intervals (CI) was applied to test the indirect and serial mediation effects of place attachment and environmental guilt (Hayes, 2017). To assess the robustness of the structural relationships, the model was subsequently re-estimated with gender, age, income, and place of residence included as demographic control variables. The stability of the focal path coefficients and the explained variance (R^2^) of the endogenous constructs were compared with those of the original model. Given the cross-sectional design, an alternative structural ordering in which environmental guilt preceded place attachment was also estimated as a competing model to assess whether the theoretically specified PA → EG ordering could be empirically distinguished from the reverse ordering. Finally, multi-group analysis (MGA) was conducted to examine whether the overall pattern of structural relationships differed across regional and social-norm groups. The omnibus structural-weights comparison was used as the primary test of H10 and H11. After establishing measurement invariance, individual structural paths were additionally examined using the AMOS critical-ratio-for-differences procedure as exploratory follow-up analyses to identify possible localized sources of between-group variation.

## 5 Results

### 5.1 Descriptive statistics and normality assessment

Table 3 presents the descriptive statistics of the study constructs. Mean scores ranged from 4.091 to 5.346, with standard deviations ranging from 0.939 to 1.297. Skewness values ranged from −0.755 to −0.331, and kurtosis values ranged from −0.221 to 1.079, indicating no substantial departures from univariate normality. However, the assessment of multivariate normality indicated a departure from multivariate normality, with a multivariate kurtosis value of 148.287 and a critical ratio (C.R.) of 79.869. Given this departure, the results were interpreted with caution, and bias-corrected bootstrapping with 5,000 resamples and 95% confidence intervals was used for inference regarding the indirect and serial mediation effects.

**Table 3:** Descriptive statistics of the constructs.

Construct	*N*	Min	Max	Mean	SD	Skewness	Kurtosis
BA	926	1	7	4.091	1.297	−0.331	−0.221
PA	926	1	7	4.759	1.254	−0.478	0.066
EG	926	1	7	5.346	0.939	−0.474	0.998
SN	926	1	7	4.733	1.180	−0.494	0.213
SBI	926	1	7	5.345	1.093	−0.755	1.079

BA, bamboo anthropomorphism; PA, place attachment; EG, environmental guilt; SN, social norms; SBI, sustainable behavioral intention; SD, standard deviation.

### 5.2 Common method bias

Because all focal variables were measured using self-reported responses collected from the same respondents within a single cross-sectional questionnaire, potential common method bias (CMB) was assessed using an unmeasured latent method construct (ULMC) approach (Podsakoff et al., 2012). Specifically, an additional latent common method factor was introduced into the original measurement model, with all measurement items loading simultaneously on their corresponding substantive constructs and on the common method factor. To obtain a parsimonious and stable model specification, the method-factor loadings were constrained to be equal, and the common method factor was specified as orthogonal to the substantive latent constructs. The resulting constrained ULMC model was then compared with the original measurement model.

As shown in Table 4, the original measurement model demonstrated acceptable fit (CMIN/DF = 4.208, RMSEA = 0.059, SRMR = 0.038, NFI = 0.938, CFI = 0.952, and TLI = 0.945). After the common latent method factor was incorporated, the constrained ULMC model also demonstrated acceptable fit (CMIN/DF = 3.961, RMSEA = 0.057, SRMR = 0.030, NFI = 0.942, CFI = 0.956, and TLI = 0.949). Compared with the original measurement model, the improvement in model fit was limited, with ΔCFI = +0.004, ΔTLI = +0.004, ΔNFI = +0.004, ΔRMSEA = −0.002, and ΔSRMR = −0.008.

**Table 4:** Results of the unmeasured latent method construct (ULMC) test.

Model	CMIN/DF	RMSEA	SRMR	NFI	CFI	TLI
Original measurement model	4.208	0.059	0.038	0.938	0.952	0.945
ULMC model	3.961	0.057	0.030	0.942	0.956	0.949
Change (Δ)	−0.247	−0.002	−0.008	+0.004	+0.004	+0.004

These results indicate that incorporating a common latent method factor produced only limited improvement in overall model fit, suggesting that common method variance is unlikely to substantially account for the observed measurement relationships. Nevertheless, this statistical assessment does not establish the absence of common method bias. Given the single-source, self-report, and cross-sectional nature of the data, potential common method bias cannot be completely ruled out and is therefore considered when interpreting the findings.

### 5.3 Reliability and validity testing

First, this study assessed the questionnaire's internal consistency using Cronbach's α. As shown in Table 1, Cronbach's α values for the five constructs ranged from 0.815 to 0.931, with an overall value of 0.913, indicating satisfactory internal consistency.

Second, exploratory factor analysis (EFA) of the 22 items was performed using SPSS 27.0. The results showed that the data were suitable for factor analysis (KMO = 0.912; Bartlett's test, *p* < 0.001). Five factors were extracted by EFA, and the total variance explained was 73.32%. Varimax rotation converged in six iterations, and all items loaded onto their corresponding factors. Thus, the EFA results were consistent with the proposed five-factor structure.

Third, confirmatory factor analysis (CFA) of the 22 measurement items was conducted using AMOS 26.0. The measurement model demonstrated an acceptable overall fit to the data: RMSEA = 0.059, SRMR = 0.038, CMIN/DF = 4.208, GFI = 0.918, AGFI = 0.896, NFI = 0.938, IFI = 0.952, CFI = 0.952, and TLI = 0.945. Although not every index met the more stringent guidelines (Hu and Bentler, 1999), the RMSEA was below 0.06, the CFI and IFI were above 0.95, and the remaining indices collectively indicated an acceptable overall fit.

Furthermore, the reliability and validity of the measurement model were assessed. As shown in Table 1, CR values ranged from 0.818 to 0.930, and the AVE for each construct ranged from 0.564 to 0.771, supporting adequate convergent validity. Standardized factor loadings ranged from 0.668 to 0.908. Although one item measuring environmental guilt exhibited a relatively lower factor loading (0.668), the loading was still within an acceptable range for retention (Hair et al., 2019). Given its theoretical importance and its role in capturing the full scope of the environmental guilt construct, this item was retained.

Finally, discriminant validity was assessed using both the Fornell–Larcker criterion and the Heterotrait-Monotrait Ratio (HTMT) (Henseler et al., 2015). As reported in Table 5, the square root of the AVE for each construct was greater than its corresponding inter-construct correlations (Fornell and Larcker, 1981). As shown in Table 6, all HTMT values were below the recommended threshold of 0.85, providing further evidence of adequate discriminant validity among the constructs (Henseler et al., 2015). Taken together, the results of both the Fornell–Larcker criterion and the HTMT assessment provide evidence supporting adequate discriminant validity in the measurement model.

**Table 5:** Discriminant validity assessment using the Fornell–Larcker criterion.

Construct	SN	BA	PA	EG	SBI
SN	0.775				
BA	0.458^***^	0.794			
PA	0.409^***^	0.357^***^	0.822		
EG	0.451^***^	0.389^***^	0.354^***^	0.751	
SBI	0.677^***^	0.273^***^	0.311^***^	0.491^***^	0.878

Data on the diagonal represent the square root of the AVE for each construct; ^***^ indicates *p* < 0.001.

**Table 6:** Discriminant validity assessment using the HTMT criterion.

Construct	SN	BA	PA	EG	SBI
SN	–				
BA	0.465	–			
PA	0.414	0.359	–		
EG	0.474	0.399	0.371	–	
SBI	0.683	0.277	0.318	0.503	–

### 5.4 Model fit

The structural model demonstrated an acceptable overall fit to the data: RMSEA = 0.063, SRMR = 0.036, CMIN/DF = 4.620, GFI = 0.923, AGFI = 0.900, NFI = 0.943, IFI = 0.955, CFI = 0.955, and TLI = 0.947. Although RMSEA was marginally above the 0.06 guideline and CMIN/DF was relatively high, the CFI and IFI exceeded 0.95 and the remaining indices collectively supported an acceptable overall model fit (Hu and Bentler, 1999).

### 5.5 Results of the structural equation modeling analysis

The hypothesized model's direct effects were tested using SEM in AMOS 26.0. The indirect effects of the hypothesized model were examined using the bootstrap method in AMOS 26.0, with 5,000 bootstrap samples at a 95% Confidence Interval (CI) level to obtain reliable statistical results. All path coefficients reported are standardized estimates.

#### 5.5.1 Direct effects

As shown in Table 7 and Figure 2, BA did not have a significant direct effect on SBI [β = 0.060, 95% CI (−0.014, 0.137), *p* = 0.106]; thus, H1 was not supported. BA was positively associated with PA (β = 0.357, *p* < 0.001) and EG (β = 0.300, *p* < 0.001). PA was positively associated with EG (β = 0.247, *p* < 0.001) and SBI (β = 0.142, *p* < 0.001). EG was also positively associated with SBI (β = 0.417, *p* < 0.001). H2, H3, H5, H6, and H8 were supported.

**Table 7:** Results of direct effect testing.

Hypotheses	Paths	β	S.E.	C.R.	*p*	Results
H1	BA → SBI	0.060	0.031	1.616	0.106	Not supported
H2	BA → PA	0.357	0.034	9.418	< 0.001	Supported
H3	PA → SBI	0.142	0.033	3.998	< 0.001	Supported
H5	EG → SBI	0.417	0.045	10.570	< 0.001	Supported
H6	BA → EG	0.300	0.029	7.632	< 0.001	Supported
H8	PA → EG	0.247	0.031	6.564	< 0.001	Supported

**Figure 2:**
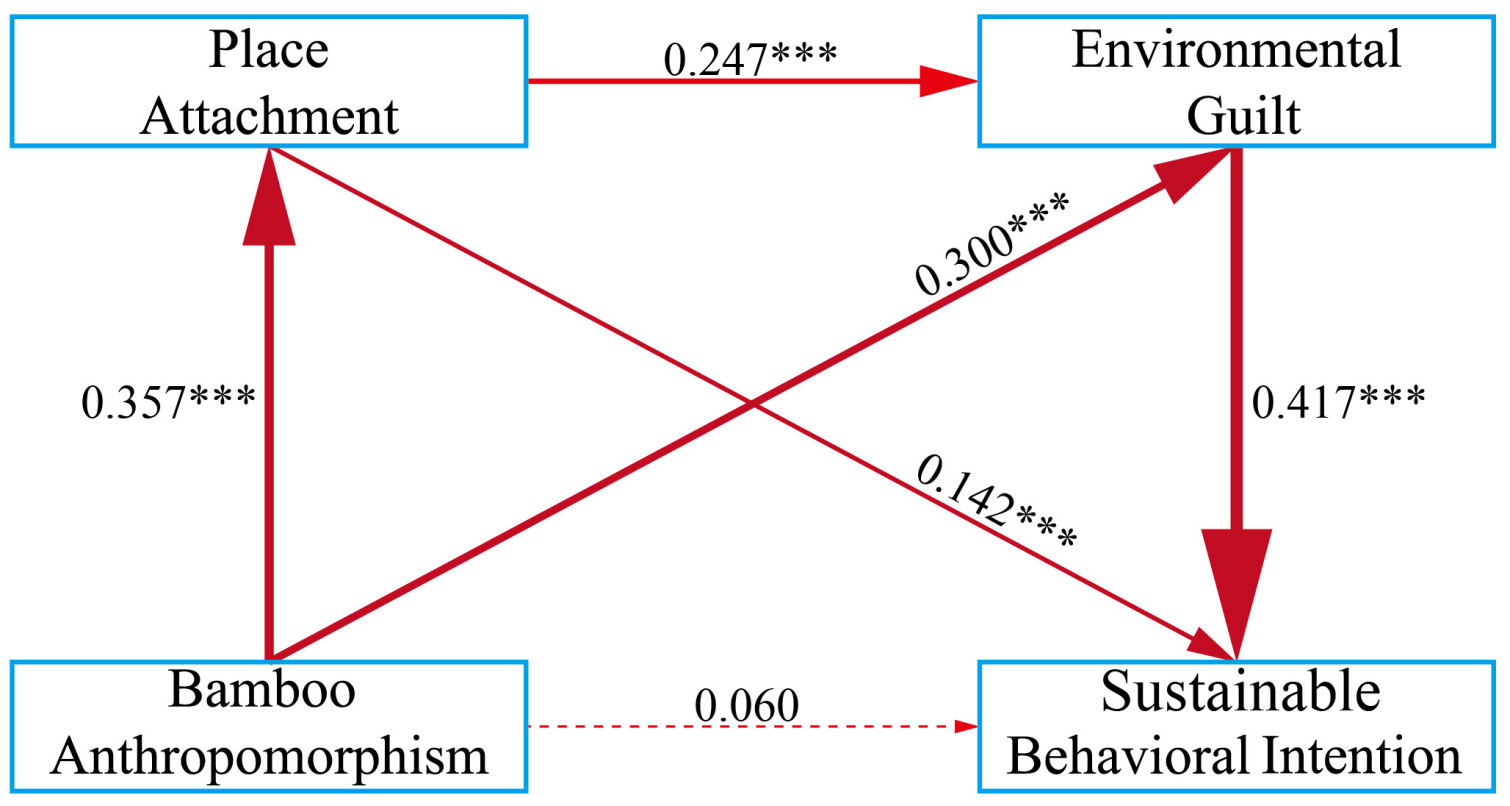
Results of the conceptual model. Solid arrows indicate statistically significant paths; dashed arrow indicates a nonsignificant path; ^***^ indicates *p* < 0.001.

#### 5.5.2 Indirect effects

As shown in Table 8, significant indirect effects were observed for the BA → PA → SBI path [β = 0.051, 95% CI (0.024, 0.084), not including 0] and the BA → EG → SBI path [β = 0.125, 95% CI (0.088, 0.171), not including 0]. A significant indirect effect was also observed for the BA → PA → EG → SBI sequential path [β = 0.037, 95% CI (0.024, 0.055), not including 0]. H4, H7, and H9 were supported.

**Table 8:** Result of indirect effect testing.

Hypotheses	Paths	β	SE	95% CI	Results
H4	Mediating path 1: BA → PA → SBI	0.051	0.015	[0.024, 0.084]	Supported
H7	Mediating path 2: BA → EG → SBI	0.125	0.021	[0.088, 0.171]	Supported
H9	Serial mediating path: BA → PA → EG → SBI	0.037	0.008	[0.024, 0.055]	Supported
	Mediating path 1–Mediating path 2	−0.075	0.029	[−0.131, −0.020]	
	Mediating path 1–Serial-mediating path	0.014	0.017	[−0.019, 0.048]	
	Mediating path 2–Serial-mediating path	0.088	0.022	[0.048, 0.135]	

Additionally, the mediating effect of environmental guilt was found to be significantly stronger than that of place attachment and the serial mediation effect. In terms of effect size, the order was as follows: the EG mediation path (Path 2), the PA mediation path (Path 1), and the serial mediation path.

#### 5.5.3 Robustness analysis with demographic controls

To assess the robustness of the structural relationships, the model was re-estimated after controlling for gender, age, income, and residence. As shown in Tables 9, 10, the inclusion of these demographic controls produced only minor changes in the estimated coefficients. The direct association between bamboo anthropomorphism and sustainable behavioral intention remained non-significant (β = 0.065, *p* = 0.080), while all other direct paths remained significant and were substantively unchanged. Similarly, the three indirect effects through place attachment, environmental guilt, and their sequential pathway remained significant, with only small changes in effect sizes. These results indicate that the main findings are robust to the inclusion of demographic controls.

**Table 9:** Comparison of direct effects with and without demographic controls.

Direct path	Original model β	*p*	Model with controls β	*p*	Conclusion
BA → SBI	0.060	0.106	0.065	0.080	Substantively unchanged
BA → PA	0.357	< 0.001	0.354	< 0.001	Substantively unchanged
PA → SBI	0.142	< 0.001	0.128	< 0.001	Substantively unchanged
EG → SBI	0.417	< 0.001	0.411	< 0.001	Substantively unchanged
BA → EG	0.300	< 0.001	0.302	< 0.001	Substantively unchanged
PA → EG	0.247	< 0.001	0.235	< 0.001	Substantively unchanged

**Table 10:** Comparison of indirect effects with and without demographic controls.

Indirect path	Original model β	95%CI	Model with controls β	95%CI	Conclusion
BA → PA → SBI	0.051	[0.024, 0.084]	0.045	[0.018, 0.078]	Substantively unchanged
BA → EG → SBI	0.125	[0.088, 0.171]	0.124	[0.088, 0.171]	Substantively unchanged
BA → PA → EG → SBI	0.037	[0.024, 0.055]	0.034	[0.021, 0.052]	Substantively unchanged

In addition to the individual path coefficients, the explanatory power of the structural model was assessed using squared multiple correlations (*R*^2^). In the original model, the explained variance was 12.78% for place attachment (PA; *R*^2^ = 0.1278), 20.41% for environmental guilt (EG; *R*^2^ = 0.2041), and 26.47% for sustainable behavioral intention (SBI; *R*^2^ = 0.2647). After controlling for gender, age, income, and residence, the corresponding *R*^2^ values increased slightly to 0.1458 (14.58%) for PA, 0.2109 (21.09%) for EG, and 0.2724 (27.24%) for SBI. These modest increases indicate that the demographic controls contributed limited additional explanatory power, while the overall explanatory performance of the structural model remained substantively unchanged.

#### 5.5.4 Alternative-ordering analysis

Given the cross-sectional nature of the data, an alternative structural model was estimated to examine whether the theoretically specified ordering between place attachment and environmental guilt could be empirically distinguished from a reversed ordering. In the proposed model, place attachment preceded environmental guilt (BA → PA → EG → SBI), whereas in the alternative model, environmental guilt preceded place attachment (BA → EG → PA → SBI). As shown in Table 11, the two models produced identical global fit indices (CMIN/DF = 4.620, RMSEA = 0.063, SRMR = 0.036, CFI = 0.955, and TLI = 0.947). Thus, the cross-sectional data did not provide an empirical basis for distinguishing the proposed PA → EG ordering from the alternative EG → PA ordering. The sequential ordering adopted in the hypothesized model should therefore be interpreted as theoretically specified rather than as evidence of an established temporal or causal sequence.

**Table 11:** Fit comparison between the proposed and alternative structural orderings.

Model	CMIN/DF	RMSEA	SRMR	CFI	TLI
Proposed model: BA → PA → EG → SBI	4.620	0.063	0.036	0.955	0.947
Alternative model: BA → EG → PA → SBI	4.620	0.063	0.036	0.955	0.947

### 5.6 Results of the multi-group analysis

#### 5.6.1 Cross-group invariance assessment

Cross-group invariance was assessed before comparing individual structural paths. A nested-model procedure was conducted separately for the regional-difference groups and the social-norm groups. The sequence comprised an unrestricted model (Model 1), a measurement-weights model in which factor loadings were constrained to equality across groups (Model 2), a structural-weights model in which structural path coefficients were constrained to equality (Model 3), and a structural-covariances model (Model 4).

Following established recommendations for assessing measurement invariance, we did not rely solely on the chi-square difference test, as it is sensitive to sample size. As shown in Table 12, invariance was evaluated using the chi-square difference test together with changes in CFI, RMSEA, SRMR, and TLI. Following Chen (2007), ΔCFI ≤ 0.010 and ΔRMSEA ≤ 0.015 were regarded as indicating negligible deterioration in model fit, with ΔSRMR used as an additional diagnostic of invariance.

**Table 12:** Results of cross-group invariance assessment.

Group	Model	Restrictions	CMIN/DF	ΔCMIN	ΔDF	RMSEA	ΔRMSEA	SRMR	ΔSRMR	CFI	ΔCFI	TLI	ΔTLI	*p*	Conclusion
Regional differences	Model 1	Unrestricted	3.044	-	-	0.047	-	0.043	-	0.949	-	0.941	-	-	-
	Model 2	Measurement weights	2.931	10.775	15	0.046	0.001	0.042	0.001	0.950	0.001	0.944	0.003	0.768	Supported
	Model 3	Structural weights	2.894	6.085	6	0.045	0	0.044	0.002	0.950	0	0.945	0.001	0.414	Supported
	Model 4	Structural covariances	2.885	0.110	1	0.045	0	0.043	0.001	0.950	0	0.945	0	0.740	Supported
Social norms	Model 1	Unrestricted	2.910	-	-	0.046	-	0.047	-	0.945	-	0.936	-	-	-
	Model 2	Measurement weights	2.850	25.328	15	0.045	0.001	0.047	0	0.944	0.001	0.938	0.002	0.046	Supported
	Model 3	Structural weights	2.832	11.324	6	0.045	0	0.054	0.007	0.943	0.001	0.938	0	0.079	Supported
	Model 4	Structural covariances	2.824	0.336	1	0.044	0.001	0.054	0	0.944	0.001	0.938	0	0.562	Supported

Δ values represent absolute changes between adjacent nested models.

For the regional groups, the measurement-weights model yielded ΔCMIN = 10.775 with ΔDF = 15 (*p* = 0.768). The corresponding changes in approximate fit indices were small (ΔRMSEA = 0.001, ΔSRMR = 0.001, ΔCFI = 0.001, and ΔTLI = 0.003), supporting metric invariance. The structural-weights model and the structural covariances model also showed no significant deterioration in fit. In summary, the results for the regional groups supported both metric invariance and overall structural-weight invariance, thereby satisfying the prerequisites for valid cross-group comparisons of the structural paths.

For the social-norm groups, the measurement-weights model yielded ΔCMIN = 25.328 with ΔDF = 15 (*p* = 0.046). Although the chi-square difference test was statistically significant, the associated changes in the approximate fit indices were small (ΔRMSEA = 0.001, ΔSRMR = 0, ΔCFI = 0.001, and ΔTLI = 0.002) and remained well within the recommended thresholds. Given the sensitivity of the chi-square difference test to sample size, the overall pattern of fit indices was taken to support metric invariance. When structural weights were constrained to equality, the chi-square difference was not statistically significant (ΔCMIN = 11.324, ΔDF = 6, *p* = 0.079). The changes in the approximate fit indices were also minimal (ΔRMSEA = 0, ΔSRMR = 0.007, ΔCFI = 0, and ΔTLI = 0) and remained within the recommended change-index thresholds (Chen, 2007), supporting overall structural-weight invariance. The structural-covariances model showed no further significant deterioration in fit (ΔCMIN = 0.336, ΔDF = 1, *p* = 0.562), with ΔRMSEA = 0.001, ΔSRMR = 0, ΔCFI = 0, and ΔTLI = 0, further supporting cross-group structural invariance.

Taken together, the results supported metric invariance across both regional and social-norm groups, thereby establishing sufficient measurement-level comparability for subsequent structural-path comparisons. Overall structural-weight invariance was also supported for both grouping variables, as constraining the structural paths to equality did not produce substantial deterioration in model fit. For the social-norm groups, although the chi-square difference test for the measurement-weights model was statistically significant, the changes in the approximate fit indices remained well within the recommended thresholds, supporting metric invariance when the full pattern of fit evidence was considered. The structural-covariances models likewise showed no substantial deterioration in fit. No latent-mean comparisons were conducted; therefore, scalar invariance was not required as a basis for the present structural-path comparisons.

#### 5.6.2 Exploratory follow-up structural path comparisons

Although the omnibus structural-weight comparisons supported overall structural invariance across both grouping variables, exploratory follow-up analyses were conducted to examine whether any individual structural paths showed localized between-group differences. These path-specific comparisons were not used as confirmatory tests of H10 or H11, but rather to identify tentative patterns warranting future hypothesis testing. Following the invariance assessment, these paths were compared across groups using the AMOS critical-ratio-for-differences procedure, with all tests based on unstandardized parameter estimates and their standard errors. Tables 13, 14 therefore report B, SE, C.R., and *p* for each group, together with the between-group *Z*-value and the unstandardized coefficient difference (ΔB) with its 95% confidence interval.

**Table 13:** Results of the multi-group analysis for northern and southern groups.

Paths	Northern (*N* = 364) B	S.E.	C.R.	*p*	Southern (*N* = 562) B	S.E.	C.R.	*p*	*Z*-value	ΔB [95% CI]
BA → SBI	0.040	0.043	0.943	0.346	0.054	0.039	1.364	0.173	−0.236	−0.014 [−0.128, 0.100]
BA → PA	0.309	0.054	5.744	< 0.001	0.346	0.043	7.965	< 0.001	−0.529	−0.037 [−0.172, 0.098]
BA → EG	0.184	0.040	4.554	< 0.001	0.227	0.036	6.382	< 0.001	−0.803	−0.043 [−0.148, 0.062]
PA → EG	0.189	0.042	4.498	< 0.001	0.166	0.036	4.633	< 0.001	0.411	0.023 [−0.085, 0.131]
PA → SBI	0.090	0.044	2.022	0.043	0.136	0.039	3.468	< 0.001	−0.790	−0.046 [−0.161, 0.069]
EG → SBI	0.598	0.074	8.076	< 0.001	0.401	0.057	7.034	< 0.001	2.118	0.197 [0.014, 0.380]

ΔB represents the difference in unstandardized coefficients between the Northern and Southern groups (northern – southern). The *Z*-value follows the same contrast direction.

**Table 14:** Results of the multi-group analysis for high and low social norms groups.

Paths	High-social-norms (*N* = 446) B	S.E.	C.R.	*p*	Low-social-norms (*N* = 480) B	S.E.	C.R.	*p*	*Z*-value	ΔB [95% CI]
BA → SBI	0.008	0.033	0.258	0.796	−0.010	0.047	−0.220	0.826	0.328	0.018 [−0.095, 0.131]
BA → PA	0.456	0.061	7.531	< 0.001	0.198	0.059	3.388	< 0.001	3.056	0.258 [0.092, 0.424]
BA → EG	0.168	0.039	4.335	< 0.001	0.201	0.044	4.530	< 0.001	−0.563	−0.033 [−0.148, 0.082]
PA → EG	0.126	0.033	3.826	< 0.001	0.132	0.037	3.595	< 0.001	−0.124	−0.006 [−0.103, 0.091]
PA → SBI	0.065	0.028	2.304	0.021	0.042	0.039	1.068	0.286	0.478	0.023 [−0.071, 0.117]
EG → SBI	0.361	0.057	6.379	< 0.001	0.347	0.058	5.974	< 0.001	0.163	0.014 [−0.145, 0.173]

ΔB represents the difference in unstandardized coefficients between the high and low social norms groups (high – low). The *Z*-value follows the same contrast direction.

For the regional groups, the omnibus structural-weights comparison supported overall structural invariance (ΔCMIN = 6.085, ΔDF = 6, *p* = 0.414). In the exploratory follow-up analysis (Table 13), the EG → SBI path yielded a nominal between-group difference (*Z* = 2.118). The unstandardized coefficient was larger in the Northern group (*B* = 0.598, SE = 0.074) than in the Southern group (*B* = 0.401, SE = 0.057), with ΔB = 0.197 and a 95% CI of (0.014, 0.380). Because the omnibus test supported structural invariance and the path-specific comparisons were not adjusted for multiplicity, this finding is interpreted as a tentative localized pattern rather than confirmatory evidence of regional moderation.

Because social norms were strongly associated with SBI and were dichotomized for the multi-group analysis, the distribution of SBI was also examined across the two social-norm groups as a descriptive check. As shown in Table 15, in the low-social-norm group (*N* = 480), SBI had a mean of 4.797 (SD = 1.073; variance = 1.150; range = 1.000–7.000), whereas in the high-social-norm group (*N* = 446), the corresponding mean was 5.933 (SD = 0.758; variance = 0.575; range = 2.500–7.000). The high-social-norm group therefore exhibited both a higher mean level of SBI and a more restricted distribution. These distributional differences provide relevant context for interpreting structural comparisons involving SBI, although they do not directly account for between-group differences in other endogenous relationships.

**Table 15:** Descriptive statistics of sustainable behavioral intention by social norm group.

Group	*N*	Mean	SD	Variance	Min	Max
Low-SN	480	4.797	1.073	1.150	1.000	7.000
High-SN	446	5.933	0.758	0.575	2.500	7.000

For the social-norm groups, the omnibus structural-weights comparison also supported overall structural invariance (ΔCMIN = 11.324, ΔDF = 6, *p* = 0.079), with negligible changes in the approximate fit indices. In the exploratory follow-up analysis (Table 14), the BA → PA path yielded a nominal between-group difference (*Z* = 3.056). The unstandardized coefficient was larger in the high-social-norm group (*B* = 0.456, SE = 0.061) than in the low-social-norm group (*B* = 0.198, SE = 0.059), with ΔB = 0.258 and a 95% CI of (0.092, 0.424). However, because this comparison was one of multiple unadjusted follow-up tests and social norms were dichotomized from an originally continuous construct, the BA → PA difference is interpreted as exploratory evidence of possible localized group-based heterogeneity rather than confirmatory evidence that social norms moderate this pathway.

Taken together, the omnibus model-level comparisons did not support H10 or H11, because overall structural-weight invariance was retained across both regional and social-norm groups. The subsequent exploratory follow-up analyses nevertheless identified two localized nominal differences, involving the EG → SBI path across regional groups and the BA → PA path across social-norm groups. These findings are treated as hypothesis-generating patterns rather than as evidence supporting H10 or H11.

## 6 Discussion

This study proposed an integrative cognition-affect-intention mechanism to examine the associations among bamboo anthropomorphism, place attachment, environmental guilt, and sustainable behavioral intention. Drawing on the Hierarchy-of-Effects model, bamboo anthropomorphism was positioned as the theoretically specified cognitive starting point, while place attachment and environmental guilt represented two hypothesized intermediate variables. Regional differences and social norms were further incorporated as contextual grouping variables.

The SEM results supported most of the hypothesized associations. Bamboo anthropomorphism was not directly associated with sustainable behavioral intention, whereas significant indirect associations were observed through place attachment, environmental guilt, and the theoretically specified sequential pathway. Environmental guilt accounted for the largest indirect effect. Given the cross-sectional design, these pathways should be interpreted as theoretically ordered statistical associations rather than evidence of causal or temporal processes.

The MGA results indicated overall structural stability across both regional and social-norm groups; therefore, H10 and H11 were not supported at the omnibus model level. Exploratory follow-up comparisons nevertheless suggested two localized patterns: a possible regional difference in the EG → SBI relationship and a possible social-norm-group difference in the BA → PA relationship. These path-specific findings should be interpreted as tentative and hypothesis-generating rather than as confirmatory evidence of moderation.

### 6.1 Findings of SEM

The first aim of this study was to examine how bamboo anthropomorphism is associated with the public's sustainable behavioral intention toward bamboo as a substitute for plastic in China. The results revealed a pattern of indirect associations consistent with the proposed psychological mechanism: bamboo anthropomorphism was not significantly directly associated with sustainable behavioral intention. Instead, significant indirect effects of bamboo anthropomorphism on sustainable behavioral intention were observed via the proposed mediating mechanisms. Specifically, environmental guilt, place attachment, and their serial mediation pathway were identified. Comparative analysis of indirect effects shows that environmental guilt emerged as the strongest mediator, followed by place attachment and their joint effect.

#### 6.1.1 Direct effects

##### 6.1.1.1 The non-significant direct effect of BA on SBI

The direct effect analysis suggests that bamboo anthropomorphism is not significantly associated with sustainable behavioral intention (β = 0.060, *p* = 0.106). This result is inconsistent with previous studies (Ketron and Naletelich, 2019; Tam et al., 2013) that have found a positive relationship between anthropomorphism and the intention to engage in environmentally friendly behavior. Two tentative explanations may account for this non-significant direct effect: the effort required for behavioral substitution and the cultural familiarity of bamboo anthropomorphism.

First, differences in the effort required by the target behavior may help explain this result. Most of the earlier research on sustainable behavior has focused on relatively low-effort acts (Kyle et al., 2005), such as biodegradable trash bags or phosphate-free detergents (Tam et al., 2013). Such behaviors typically require only minor changes to existing habits. Bamboo can be used to replace plastic to demonstrate environmental awareness, but it is a high-effort behavior (Ramkissoon et al., 2013). The public may have to pay extra money and face a certain hurdle to entry. Simple anthropomorphism strategies cannot overcome the above direct barriers (Ketron and Naletelich, 2019). Anthropomorphism primarily causes emotional excitation and is a side route. However, the reasons for structural substitution behavior are generally rational, such as the need for functionality or cost. If the barriers to behavioral switching are too high, then emotional appeals will fail. Previous research has further shown that anthropomorphism can reduce consumers' repair intentions (Qin et al., 2024).

Second, the cultural context can also explain this deviation from the previous study. The participants in previous studies did not have a deep anthropomorphic cultural background (Epley et al., 2007; Lin et al., 2026; Liu et al., 2024). However, bamboo already has strong cultural connotations in Chinese culture, such as “modesty” and “integrity”, so anthropomorphic expressions are not very newsworthy or emotionally moving. Instead of creating a new association for the Chinese public, bamboo anthropomorphism should “awaken” existing meanings to raise the threshold for impact. Therefore, different cultures need different forms of contextualization or innovative narratives in China for effectiveness. This contrast shows how cultural context can fundamentally alter the success and universality of sustainable behavioral intention toward bamboo as a substitute for plastic.

Taken together, the non-significant direct effect of bamboo anthropomorphism on sustainable behavioral intention should not be interpreted as contradicting or correcting the HOE framework. In the present study, the HOE model serves as an organizing framework for distinguishing cognition, affect, and intention rather than as a requirement that cognition must exert a significant direct effect on intention. The findings instead suggest that, in the context of bamboo as a substitute for plastic, the behavioral relevance of anthropomorphic cognition is primarily transmitted through affective and moral mechanisms, particularly place attachment and environmental guilt.

##### 6.1.1.2 The direct associations of place attachment and environmental guilt on SBI

Place attachment was positively associated with SBI (β = 0.142), in line with the results of Daryanto and Song (2021), Soopramanien et al. (2023), and Qiu et al. (2025). In the context of bamboo-based plastic substitution, giving bamboo local symbolic significance will help people feel a sense of identity and long-term interest. Although place attachment showed a significant positive association with SBI, the association between environmental guilt and SBI was substantially stronger. As shown in the previous research, place attachment can inspire people to behave in a more environmentally friendly way. However, such behavior usually needs support from moral guidance or social norms (Daryanto and Song, 2021).

Environmental guilt was also positively associated with SBI (β = 0.417). Prior research shows that guilt involves self-reproach and may motivate compensatory behavior (Raffone et al., 2025). One possible interpretation is that anthropomorphic representations of bamboo may increase the salience of environmental responsibility.

In short, this study clarifies the relative strength of affective and moral factors within the integrative cognitive mechanism underlying sustainable behavioral intention. Environmental guilt showed a stronger association with sustainable behavioral intention than place attachment did.

#### 6.1.2 Indirect effects

##### 6.1.2.1 Affective pathways: the indirect-only mediation role of place attachment

The affective pathway through place attachment showed a significant indirect association between bamboo anthropomorphism and sustainable behavioral intention (β = 0.051, 95% CI excluding 0). Bamboo anthropomorphism was positively associated with place attachment (β = 0.357, *p* < 0.001), which was in turn positively associated with sustainable behavioral intention. Because the indirect effect was significant whereas the direct BA → SBI path was not [β = 0.060, 95% CI (−0.014, 0.137), *p* = 0.106], this pattern is consistent with indirect-only mediation (Zhao et al., 2010; Rucker et al., 2011). This finding highlights the potential relevance of place-based emotional bonds in sustainable behavioral intention, consistent with prior research linking place attachment to pro-environmental outcomes (Daryanto and Song, 2021; Jiao et al., 2023). However, the interpretation of this pathway requires caution. The place attachment scale used in this study assesses respondents' general emotional attachment to their place of residence rather than attachment specifically elicited by bamboo, bamboo landscapes, or bamboo-for-plastic substitution. Therefore, the significant BA → PA relationship should not be interpreted as direct evidence that anthropomorphizing bamboo causes individuals to develop stronger place attachment through bamboo-related hometown or landscape associations. Rather, the results establish an association between bamboo anthropomorphism and general residential place attachment that is consistent with, but does not uniquely demonstrate, the proposed affective mechanism.

Alternative explanations are also possible. For example, broader individual dispositions such as connectedness to nature, environmental concern, place-based environmental identity, or general pro-environmental orientation may contribute to both the tendency to anthropomorphize bamboo and the tendency to report stronger place attachment. In addition, because all constructs were assessed through a cross-sectional self-report survey, shared response tendencies cannot be completely excluded. The present findings should therefore be understood as supporting a theoretically consistent indirect association rather than establishing a bamboo-specific causal psychological process.

More broadly, the findings suggest that place-based affect may constitute an important component of the proposed cognition–affect–intention framework. Research in nature tourism and conservation has similarly reported positive relationships between emotional attachment to natural places and support for conservation policies or responsible tourism (Yoon et al., 2024). Building on this literature, the present study extends anthropomorphism research by examining its association with place attachment in the specific context of bamboo as a substitute for plastic. Unlike previous studies that have primarily focused on relationships between anthropomorphism and product attitudes (Sehgal et al., 2023; Wang and Park, 2023), the present findings point to a potentially relevant connection between anthropomorphic perceptions and place-based affect. From a practical perspective, this association suggests that environmental communication could explore bamboo not only in terms of its functional and environmental attributes but also as a culturally and place-relevant symbol. However, whether anthropomorphic communication actually strengthens place attachment or sustainable behavioral intention requires further experimental or longitudinal verification.

##### 6.1.2.2 Moral pathways: the indirect-only mediation role of environmental guilt

A similar qualification applies to the environmental-guilt pathway. Environmental guilt showed a significant indirect association between bamboo anthropomorphism and sustainable behavioral intention (β = 0.125, 95% CI excluding 0), while the direct BA → SBI path remained nonsignificant [β = 0.060, 95% CI (−0.014, 0.137), *p* = 0.106], again indicating an indirect-only mediation pattern (Zhao et al., 2010; Rucker et al., 2011). However, the environmental guilt items assessed respondents' general feelings of guilt and responsibility concerning human-caused environmental damage; they did not specifically refer to plastic pollution, bamboo products, or bamboo-for-plastic substitution. Consequently, the present results indicate that bamboo anthropomorphism is associated with a broader environmental moral response, which in turn is associated with sustainable behavioral intention, but they do not establish that anthropomorphizing bamboo specifically generates guilt about plastic use. This distinction narrows the interpretation of the proposed moral pathway and highlights the need for more context-specific measurement in future research.

This study further showed that the indirect effect through environmental guilt was larger than that through place attachment. Although both variables were significantly implicated in the indirect association between bamboo anthropomorphism and sustainable behavioral intention, environmental guilt represented the stronger pathway. This finding highlights the potential importance of moral emotion within the proposed framework. In line with prior research, bamboo anthropomorphism was also positively associated with environmental guilt (Tam, 2019). A possible explanation is that anthropomorphic representations may make the environmental consequences of plastic pollution more psychologically salient, which may in turn be related to lower psychological distance and stronger pro-environmental intentions. Because the data are cross-sectional, however, this interpretation should not be taken as evidence that anthropomorphism causally produces guilt or that guilt subsequently causes sustainable behavioral intention.

##### 6.1.2.3 The serial mediation effect

The results suggest a theoretically specified serial indirect association involving both affective and moral components. In the hypothesized model, BA was positively associated with PA, PA with EG, and EG with SBI. Although the standardized serial indirect effect was small (β = 0.037), it was statistically supported. However, because all constructs were measured simultaneously, this ordering should not be interpreted as evidence of temporal precedence or a confirmed sequential psychological process.

The alternative-ordering analysis further showed that reversing the order of place attachment and environmental guilt resulted in identical model fit. This finding reinforces the need for caution in interpreting the serial indirect effect: although the PA → EG sequence is theoretically consistent with the proposed framework, the present cross-sectional data cannot establish that place attachment temporally precedes environmental guilt.

These findings should not be interpreted as correcting or rejecting the HOE model. Rather, they clarify how the broad cognition–affect–intention organization is manifested in the present empirical context. The non-significant BA → SBI path indicates that bamboo anthropomorphism was not directly associated with sustainable behavioral intention after the mediating variables were considered. Instead, significant indirect effects were observed through place attachment, environmental guilt, and their sequential pathway. Thus, for bamboo-based plastic substitution, the findings are consistent with an indirect cognition–affect–intention account in which the association between anthropomorphic cognition and sustainable behavioral intention is statistically transmitted through identifiable affective mechanisms.

### 6.2 Findings of MGA

The second research aim was to examine whether the overall pattern of structural relationships within the proposed cognition–affect–intention model differed across regional and social-norm groups. The omnibus structural-weight comparisons did not indicate significant overall structural heterogeneity for either grouping variable. Accordingly, H10 and H11 were not supported.

Despite this overall structural stability, exploratory path-specific follow-up analyses identified two localized patterns. For regional differences, the omnibus structural-weights comparison did not indicate overall structural non-invariance between the southern and northern groups. However, the path-specific critical-ratio analysis identified a localized difference in the EG → SBI relationship. The unstandardized coefficient was larger in the Northern group (*B* = 0.598) than in the Southern group (*B* = 0.401), and the critical ratio for the difference was *Z* = 2.118. This pattern suggests that environmental guilt may translate more strongly into sustainable behavioral intention among northern respondents. Because the overall structural-weights model remained invariant, however, this result should be understood as a localized path-specific difference rather than as evidence that the regional groups differ across the structural model as a whole. One possible interpretation is that differences in exposure to bamboo resources and bamboo-related practices may shape the behavioral relevance of environmental guilt. Bamboo is substantially more available and culturally embedded in many southern regions, whereas its relative scarcity in northern regions may make bamboo-based substitution more salient when environmental responsibility is activated. This interpretation remains tentative and should be verified in future research because the broader regional structural model did not differ significantly.

For the social-norm groups, the omnibus structural-weights comparison likewise supported overall structural invariance. Nevertheless, the exploratory follow-up analysis identified a nominal difference in the BA → PA path (*Z* = 3.056). The unstandardized association between bamboo anthropomorphism and place attachment was larger in the high-social-norm group (*B* = 0.456, SE = 0.061) than in the low-social-norm group (*B* = 0.198, SE = 0.059), with ΔB = 0.258 and a 95% CI of (0.092, 0.424). This pattern suggests possible group-based heterogeneity in the association between anthropomorphic perceptions of bamboo and place attachment. However, social norms were originally measured as a continuous construct and were dichotomized only for the purpose of the multi-group analysis. The high- and low-social-norm groups also differed substantially in the level and dispersion of sustainable behavioral intention. Therefore, the observed BA → PA difference should not be interpreted as confirmatory evidence of a continuous moderating effect of social norms. Instead, it provides an exploratory pattern that may inform more direct tests of social-norm interactions in future research.

Taken together, the MGA results indicate that the proposed cognition–affect–intention structure was broadly stable across both regional and social-norm groups. The two localized path differences therefore do not overturn the omnibus finding of structural invariance. Rather, they identify specific relationships—EG → SBI across regional groups and BA → PA across social-norm groups—that may warrant targeted hypothesis testing in future preregistered studies using more direct contextual measures, continuous interaction approaches, and multiplicity-adjusted inference.

### 6.3 Theoretical implications

First, this study refines the application of the HOE framework to sustainable behavioral intention by differentiating two theoretically distinct mechanisms within the affective stage. Place attachment represents a place-based relational response, whereas environmental guilt represents a self-conscious moral emotion. Their significant individual and sequential indirect effects suggest that the affective stage need not be treated as psychologically homogeneous in this context. Rather than modifying the basic cognition–affect–intention architecture of the HOE model, these findings provide a more differentiated account of the psychological processes that may operate within its affective stage.

Second, this study advances anthropomorphism cognition research by identifying an indirect-only mechanism in a relatively high-effort sustainability context. Bamboo anthropomorphism did not directly predict sustainable behavioral intention. Its association with sustainable behavioral intention was mediated by place attachment, environmental guilt, and their sequential mediation, with environmental guilt representing the strongest pathway. The findings therefore shift attention from whether anthropomorphism directly works to how it becomes consequential through identifiable psychological processes.

Third, this study provides a more cautious account of contextual heterogeneity within the cognition–affect–intention framework. The omnibus MGA results indicated that the overall pattern of structural relationships was broadly stable across both regional and social-norm groups. Thus, the present findings do not provide confirmatory evidence that these contextual variables moderate the model as a whole. Nevertheless, exploratory follow-up comparisons identified localized differences in the EG → SBI and BA → PA paths. These patterns generate more specific hypotheses regarding where contextual variation may occur within the mechanism and can be tested directly in future preregistered studies.

### 6.4 Practical implications

The practical implications of the present findings should be interpreted in proportion to the magnitude and scope of the observed effects. Bamboo anthropomorphism was not directly associated with sustainable behavioral intention, while the statistically significant associations operated indirectly through place attachment and environmental guilt. Among these pathways, the indirect association through environmental guilt was the strongest, although its magnitude remained modest. Moreover, the outcome measured in this study was behavioral intention rather than observed adoption behavior. The findings therefore provide preliminary guidance for the design and testing of sustainability communication strategies rather than direct evidence for large-scale behavioral or policy interventions.

For policymakers and environmental communicators, the results suggest that communication concerning bamboo-based alternatives may benefit from testing messages that combine factual environmental information with carefully designed affective or moral cues. In particular, because environmental guilt represented the strongest indirect pathway in the present model, communication experiments could examine whether messages that make the environmental consequences of plastic consumption salient increase willingness to consider bamboo alternatives. Such communication should avoid excessive guilt appeals and should be evaluated empirically for both effectiveness and possible unintended responses.

Place-related communication may also warrant further investigation, although the corresponding indirect effect was smaller and the place attachment measure used in the present study was not bamboo-specific. Therefore, rather than recommending place-branding programs on the basis of the present findings, future pilot studies could test whether locally relevant bamboo narratives or place-based environmental messages increase engagement with bamboo alternatives in settings where bamboo has genuine ecological or cultural relevance.

The findings concerning social norms and regional heterogeneity may similarly inform the targeting of future communication research, but they should not be interpreted as evidence for immediate differentiated policy implementation. Small-scale field experiments or pilot communication programs could test whether making existing sustainable practices socially visible improves intentions and, more importantly, actual adoption behavior across different contexts.

For industry associations and enterprises, the present results suggest that anthropomorphic representations of bamboo should not be expected to generate behavioral adoption on their own. Communication strategies may instead be evaluated as one component of a broader intervention that also addresses practical barriers such as product price, availability, functionality, and convenience. Firms could use controlled message testing, A/B experiments, or limited field trials to examine whether anthropomorphic, environmental, or normative messages improve actual product choice relative to conventional information-based communication.

Overall, the present study supports the testing of targeted communication approaches rather than the immediate implementation of large-scale certification, subsidy, branding, or demonstration programs. Before resource-intensive interventions are considered, future research should establish whether the observed intention-based relationships translate into actual consumer behavior and whether the resulting behavioral effects are sufficiently large to justify implementation costs.

### 6.5 Limitations and future research

This study used cross-sectional survey data from 926 respondents across 31 provincial-level administrative regions in China to provide an initial examination of the associations among bamboo anthropomorphism, place attachment, environmental guilt, and sustainable behavioral intention toward bamboo as a substitute for plastic. Although the findings provide preliminary support for the proposed integrative cognition–affect–intention framework, several limitations should be acknowledged, particularly those related to the cross-sectional design and the interpretation of the hypothesized indirect pathways.

First, the operationalization of regional context is relatively coarse. The present study divided respondents into northern and southern groups using the Qinling–Huai River boundary. Although this classification provides a useful broad geographic comparison and corresponds approximately to major differences in the spatial distribution of bamboo resources, it is a coarse contextual indicator and cannot isolate bamboo-resource abundance from the many other socioeconomic, cultural, environmental, industrial, and policy differences between northern and southern China. Moreover, attributing aggregate regional characteristics directly to individual psychological processes would risk an ecological fallacy. The present regional MGA should therefore be interpreted as evidence of broad regional heterogeneity rather than as a direct test of the effect of bamboo-resource endowment. Future studies should replace or complement the binary North–South classification with direct province-level contextual indicators, such as bamboo forest area, bamboo forest coverage, bamboo-industry output value, bamboo-product production, or related measures of market and industrial exposure. These continuous province-level variables could be linked to individual respondents according to their province of residence and analyzed using a multilevel modeling framework, with individuals nested within provinces. Such a design would distinguish individual-level psychological relationships from province-level contextual effects and would allow cross-level interactions to test more directly whether bamboo-resource abundance or bamboo-industry development moderates the relationships among bamboo anthropomorphism, affective responses, and sustainable behavioral intention.

Second, the demographic composition of the sample should be considered when interpreting the findings. The sample was recruited through an online questionnaire platform rather than through a probability-based nationally representative sampling design, and no post-stratification weighting was applied. Women constituted 62.74% of the sample, and 93.09% of respondents were between 18 and 45 years of age, resulting in an overrepresentation of female and younger/middle-aged respondents relative to the general population. Therefore, the findings should primarily be interpreted as evidence concerning the relationships among the study constructs within the surveyed sample rather than as population-weighted estimates of attitudes or behavioral intentions in the broader Chinese population. Future research could employ probability-based sampling, demographic quotas, or post-stratification weighting to improve population representativeness.

Third, the potential influence of common method bias. All focal constructs were measured through self-reports from the same respondents using a single questionnaire administered within the same survey period. Although the constrained ULMC analysis showed that introducing a common latent method factor resulted in only limited improvement in model fit, statistical controls cannot completely eliminate concerns about common method bias. This issue may be particularly relevant to environmental guilt, and sustainable behavioral intention because both were assessed within the same instrument and in close measurement proximity. Future research should therefore incorporate stronger procedural and design-based remedies, such as temporal separation of predictor, mediator, and outcome measurements, multi-source data, longitudinal or experimental designs, and theoretically unrelated marker variables. Such approaches would provide a more rigorous assessment of whether the observed relationships remain robust when common method influences are reduced.

Fourth, the mediating measures have some contextual limitations. Although bamboo anthropomorphism was measured specifically in relation to bamboo, the place attachment scale assessed general attachment to respondents' place of residence, and the environmental guilt scale assessed general guilt concerning human-caused environmental damage. Thus, the present study cannot determine whether the observed indirect associations operate specifically through bamboo-related place attachment or bamboo/plastic-related environmental guilt. Future research should develop or adapt context-specific measures that explicitly connect place attachment to bamboo-related landscapes, cultural settings, or local bamboo environments, and environmental guilt to plastic consumption, plastic pollution, and bamboo-for-plastic substitution. Such measures would provide a more direct test of the proposed psychological mechanism.

Fifth, although the robustness analysis controlled for available demographic variables, other relevant individual-level factors, such as environmental knowledge, prior experience with bamboo products, price sensitivity, and education, were not measured. Future research should incorporate these variables to further assess the robustness and explanatory power of the proposed relationships.

Finally, the path-specific MGA involved 12 follow-up comparisons across the two grouping variables. These comparisons were not adjusted for multiplicity, and the two observed differences were relatively close to the conventional significance threshold. Although unstandardized coefficient differences and 95% confidence intervals are reported to improve transparency, the path-specific findings should be regarded as exploratory. Future studies should preregister specific cross-group path hypotheses and use multiplicity-adjusted inference or independent samples to confirm these patterns.

## 7 Conclusions

This study examined the relationships among bamboo anthropomorphism, place attachment, environmental guilt, and sustainable behavioral intention toward bamboo as a substitute for plastic in China within an integrative cognition–affect–intention framework derived from the Hierarchy-of-Effects model. Based on survey data from 926 respondents across 31 provincial-level administrative regions, the findings provide evidence that the relationship between bamboo anthropomorphism and sustainable behavioral intention is primarily indirect rather than direct.

The SEM findings showed that bamboo anthropomorphism was not significantly directly associated with sustainable behavioral intention. Instead, both place attachment and environmental guilt showed indirect-only mediation in the association between bamboo anthropomorphism and sustainable behavioral intention. Among these pathways, environmental guilt showed the largest indirect effect. These findings suggest that the potential behavioral relevance of anthropomorphic cognition may lie less in anthropomorphism alone than in its association with relational affect and environmental moral emotion. At the same time, because place attachment and environmental guilt were measured as general rather than bamboo-specific constructs, the results should be understood as supporting theoretically consistent indirect associations rather than demonstrating a bamboo-specific psychological process.

The multi-group analyses supported overall structural stability across both regional and social-norm groups, and the omnibus tests therefore did not support H10 or H11. Exploratory follow-up comparisons nevertheless suggested a localized regional difference in the EG → SBI relationship and a localized social-norm-group difference in the BA → PA relationship. Because these comparisons were not adjusted for multiplicity and the social-norm analysis involved dichotomization of a continuous construct, these findings should be considered hypothesis-generating rather than confirmatory evidence of moderation.

Overall, this study extends research on sustainable material substitution by distinguishing relational place-based affect from self-conscious environmental moral emotion within a cognition–affect–intention framework. The results highlight environmental guilt and, to a lesser extent, place attachment as potentially relevant psychological correlates of sustainable behavioral intention toward bamboo-based alternatives. From an applied perspective, the findings support further testing of communication approaches that combine information about bamboo with carefully designed affective or environmental-responsibility cues, rather than immediate large-scale policy or behavioral interventions.

These conclusions should be interpreted within the limits of the study design. All focal constructs were measured through a single cross-sectional self-report survey, the outcome concerned behavioral intention rather than observed behavior, the sample was not nationally representative, and the proposed mediating sequence reflects a theoretically specified statistical ordering rather than an empirically established temporal process. Longitudinal, experimental, and behavior-based research using more context-specific measures is therefore required before causal mechanisms or broad policy implications can be established.

## Data Availability

The original contributions presented in the study are included in the article/supplementary material, further inquiries can be directed to the corresponding author.
